# Reply to Alt et al. Comment on “Lunz et al. Impact and Modification of the New PJI-TNM Classification for Periprosthetic Joint Infections. *J. Clin. Med.* 2023, *12*, 1262”

**DOI:** 10.3390/jcm12216846

**Published:** 2023-10-30

**Authors:** Andre Lunz, Burkhard Lehner, Moritz N. Voss, Kevin Knappe, Sebastian Jaeger, Moritz M. Innmann, Tobias Renkawitz, Georg W. Omlor

**Affiliations:** 1 Department of Orthopaedics, Heidelberg University Hospital, Schlierbacher Landstrasse 200a, 69118 Heidelberg, Germanytobias.renkawitz@med.uni-heidelberg.de (T.R.);; 2 Center for Orthopedics and Joint Replacement, Marienhaus Hospital St. Wendel—Ottweiler, Am Hirschberg 1, 66606 St. Wendel, Germany

We greatly appreciate the comments made by Alt et al. [1] regarding our recently published article in *JCM*, in which we report the impact and modification of a new classification (PJI-TNM classification) for periprosthetic joint infections (PJI) [2]. Alt et al. defined the soft tissue situation (T = tissue and implant), the causative microorganism (N = non-human cell), and the host (M = comorbidity of the patient) as the key characteristics of a PJI and transferred them to the three-letter backbone of TNM [3,4,5].

We acknowledge that while our research established significant correlations between the PJI-TNM classification and certain surgical and clinical outcome parameters, there are additional very important variables, such as reinfection rates or the need for surgical revision after second-stage surgery, which warrant investigation in future studies. Revealing a significant correlation with these parameters would strongly underline the great benefit of utilizing the new PJI-TNM classification in clinical practice. Nevertheless, our study is based on a consecutive series of patients and our findings have already added significant value to the practical applications of the PJI-TNM classification. As mentioned by Alt et al., the significant correlation of the “M-status” with mortality, for example, reiterates the clinical relevance of our efforts. We concur with the viewpoint that the complexity of PJIs necessitates a comprehensive classification system to derive accurate treatment recommendations and to enable precise scientific assessment. The evolution of the TNM classification in oncology over the past four decades is indeed a testament to the idea that classifications can and should undergo iterative refinement based on evidence and clinical feedback [6,7].

The point regarding the use of the Charlson Comorbidity Index (CCI) vs. the use of the American Society of Anesthesiologists (ASA) classification for assessing the “M-status” of patients is well-taken [8,9]. Although the ASA classification is widely used and collected in most arthroplasty registries, including the German Arthroplasty Registry (ERPD), we respect the evidence that Alt et al. has provided regarding the significant correlation of the CCI with mortality in musculoskeletal infections and orthopaedic surgery [10]. The development of the “PJI-TNM App” for mobile devices is an admirable step forward in facilitating clinical use. We appreciate the effort put into this initiative and will certainly consider its utility in our future studies and recommendations.

We have proposed a separate “p-status” referring exclusively to the type of infected prosthesis and proposed a new version of the classification, namely, the PJI-pTNM classification. We are pleased by the positive acknowledgment by Alt et al. of our proposal to classify megaprostheses separately, given their distinctive challenges in treatment.

Regarding our modified T-status, we appreciate the concerns described in the comment by Alt et al. about categorizing both the presence of fistulae and significant soft tissue defects on the same level (T2). We understand the distinction between the two, particularly given the varied management approaches and outcomes associated with them. Our intention was to explore potential correlations and provide a comprehensive but simple classification; however, we absolutely acknowledge the clinical differences and will certainly revisit this aspect. Finally, we are pleased to note the positive feedback regarding our simplified N-status and its resultant correlations.

In conclusion, we remain committed to further research on the PJI-(p)TNM classification as well as other topics regarding the prevention, diagnosis, and therapy of PJIs. Collaboration and open dialogue between research groups are crucial for the evolution of scientific knowledge. We would sincerely appreciate an opportunity for collaboration or further discussion, be it through PJI research, workshops, or symposiums. Once again, we would like to thank Alt et al. for the thorough and constructive feedback.
